# Biotic Interactions Overrule Plant Responses to Climate, Depending on the Species' Biogeography

**DOI:** 10.1371/journal.pone.0111023

**Published:** 2014-10-30

**Authors:** Astrid Welk, Erik Welk, Helge Bruelheide

**Affiliations:** 1 Institute of Biology/Geobotany and Botanical Garden, Martin Luther University Halle-Wittenberg, Halle (Saale), Germany; 2 German Centre for Integrative Biodiversity Research (iDiv) Halle-Jena-Leipzig, Leipzig, Germany; York U, Canada

## Abstract

This study presents an experimental approach to assess the relative importance of climatic and biotic factors as determinants of species' geographical distributions. We asked to what extent responses of grassland plant species to biotic interactions vary with climate, and to what degree this variation depends on the species' biogeography. Using a gradient from oceanic to continental climate represented by nine common garden transplant sites in Germany, we experimentally tested whether congeneric grassland species of different geographic distribution (oceanic *vs*. continental plant range type) responded differently to combinations of climate, competition and mollusc herbivory. We found the relative importance of biotic interactions and climate to vary between the different components of plant performance. While survival and plant height increased with precipitation, temperature had no effect on plant performance. Additionally, species with continental plant range type increased their growth in more benign climatic conditions, while those with oceanic range type were largely unable to take a similar advantage of better climatic conditions. Competition generally caused strong reductions of aboveground biomass and growth. In contrast, herbivory had minor effects on survival and growth. Against expectation, these negative effects of competition and herbivory were not mitigated under more stressful continental climate conditions. In conclusion we suggest variation in relative importance of climate and biotic interactions on broader scales, mediated via species-specific sensitivities and factor-specific response patterns. Our results have important implications for species distribution models, as they emphasize the large-scale impact of biotic interactions on plant distribution patterns and the necessity to take plant range types into account.

## Introduction

Understanding the causes of species geographical distributions is a major research goal in ecology, often driven by the desire to model future species distributions in a world undergoing climate change. Soberón [1] summarized the main determinants of species distribution, which apart from a species' dispersal capacity comprise the physiological niche and biotic interactions. Depending on the species' physiological niche [2], [3], a fundamental range of environmental, and in particular climatic conditions, defines the suitable range for growth, reproduction and establishment of populations [4]. This fundamental range of environmental conditions is usually modified by biotic interactions, for example owing to constricted tolerances in the presence of competitors or herbivores [1], [5]. The result is the species' ecological [2], [3]or realized niche [6], which is the environmental range of conditions under which a species does occur in nature.

Correlative species distribution models are based on the central assumption that on broad geographic scales, species' spatial distributions are in equilibrium with climate, while biotic interactions are of minor importance [7]. At the same time, is has been emphasized that biotic interactions are important at local scales, e.g. for presence and abundance in communities [1], [8]. However, it has been recognized that detrimental biotic interactions, such as competition and herbivory, have the potential to limit plant distribution also on large spatial scales [9], [10]. For instance, Bruelheide & Scheidel [11] demonstrated that the altitudinal distribution of a montane plant species is restricted to higher elevations because of increasing slug herbivory in the lowlands.

Impacts of biotic interactions on plant performance have been demonstrated to change along climate gradients as for example was proposed by the stress gradient hypothesis for competition [12], [13]. Thereby, competition should gain in relative importance under benign climate conditions. For example, Loehle [14] suggested that the northern range limits of North American tree species are limited by cold tolerance, while competitive ability should determine the southern range limits. This has also been demonstrated with examples of increasing competition with decreasing altitude [15]. Similarly, other biotic interactions might vary with climate conditions. For example, slug herbivore pressure was shown to increase with decreasing altitude [16].

In addition to altitudinal and latitudinal climate gradients, environments in Europe are also structured by a distinct longitudinal differentiation in climate. A strong gradient exists from oceanic climate in Western Europe, with relatively narrow annual temperature ranges and constantly humid conditions, to continental climate in Eastern Europe, with large temperature seasonality and low annual precipitation. Along this gradient, the general physiological growth conditions for plants get harsher with increasing continentality. The strong impact of this continentality gradient on plant distribution is also evident in phytogeographical classification systems that classify plant species according to their geographic distribution along this gradient into oceanic and continental plant range types [17], [18].

Species populations are expected to increase in performance, the closer their locations' growth conditions is to the species' climate optimum [19]. Under the general assumption that species' geographic distributions reflect their environmental requirements, species should do better under conditions which are climatically less peripheral. Testing this assumption for North American tree species, Purves [20] found distinct differences in growth and demographic rates between northern and southern peripheral range sections when compared with the core area. Accordingly, oceanic and continental plant species should perform better in oceanic and continental climates respectively. On the other hand, this view has recently been challenged as glasshouse experiments revealed no clear differences between responses of oceanic and continental species in respect to soil moisture levels and frost hardiness [21], [22]. However, multiple species field tests on plant range type-specific adaptations are still missing.

If closely related plant species tend to be similar in their fundamental niche requirements, differences in geographical distribution patterns might be caused by dispersal limitation, biogeographical history and biotic interactions. Differences in biotic characteristics are probably responsible for the large amount of unexplained interspecific variation in periphery-core comparisons of species performance [20]. As predicted by the stress-gradient-hypothesis, plant-plant interactions can turn from competition to facilitation with increasing abiotic stress [15]. Thus, competition should be less intense in continental compared to oceanic regions. Similarly, mollusc herbivory might also be reduced in continental regions as slugs (e.g. Arionidae) show clear preferences to more benign climate conditions [16]. Hence, species distributed in continental regions may be less adapted to negative biotic interactions and more vulnerable to biotic stress. This mismatch might be disadvantageous in the climatically more favourable oceanic regions. In consequence, detrimental biotic interactions should have a different impact depending on the species' plant range type.

**Figure 1:**
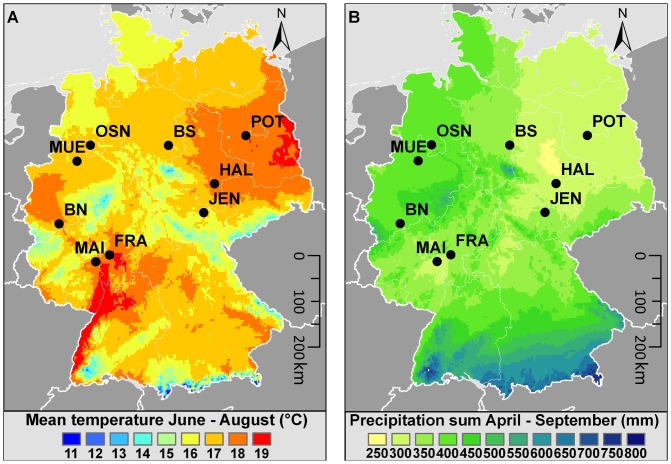
Locations of the Botanical Gardens in the transplant experiment showing the main gradients in climatic differences. A) Sum of the monthly mean temperature in summer (June-August), B) Precipitation of the vegetation period in mm. Climate data were obtainded from [26] and refer to the same periods as used in Table 1, but refer to long-term averages. For abbreviations of locations see Table 1.

Based on these considerations a transplant experiment was set up in nine Botanic Gardens along a continentality gradient in Germany (Fig. 1), where the relative impact of biotic interactions (competition and mollusc herbivory) and climate was tested with congeneric plant species of contrasting plant range types. Such transplant experiments have been used before and demonstrated a strong climatic impact on the transplants' survival, growth and reproduction [23]–[25].

The following hypotheses were tested: H1) There is an interactive effect between climate and biotic treatments on plant performance. In particular, we expected the effect of competition and herbivory to become weaker with increasing climatic continentality. H2) At the oceanic end of the gradient, species with general oceanic distribution should perform better than species with general continental distribution range and vice versa, indicating range type-specific adaptation. H3) Depending on the plant range type, biotic treatments affect species differently, as the continental plant species should be more susceptible to competition and herbivory. Furthermore, assuming that the relative importance of herbivory and competition decreases with increasingly continental climate, the negative biotic effects on continental species should decrease with increasing continentality. Testing these hypotheses aims at improving the mechanistic understanding of species distribution patterns.

## Material and Methods

### Ethics statement

The authorities that issued the permit to use the Botanical Gardens were the scientific or technical directors of the gardens. As they also assigned the piece of land to us, carrying out the experiment without this permit would not have been possible. Seeds for the experiment were collected from public land and all regulations concerning protected or endangered species were respected.

### Climate gradient

To establish a climatic gradient for common garden locations, we made use of the network of Botanical Gardens, as they provide excellent conditions for reproducibility, and generally have similar soil conditions, i.e. fertile garden soils (hortisols). Additionally, these gardens usually suffer from high mollusc densities, which motivated us to manipulate mollusc herbivory as a negative biotic impact. Using the geographic coordinates of 66 major Botanical Gardens in Germany we extracted the mean values for mean minimum temperatures of the coldest month and mean annual precipitation for the last 50 years, using the WORLDCLIM dataset [26]. We chose 12 gardens located along a gradient from oceanic (mild winter and high precipitation) to continental (cold winter and low precipitation) climate conditions. Out of these, nine gardens responded positively to our request for conducting an experiment (Fig. 1, Table 1).

**Table 1:** Climatic conditions of the Botanical Gardens and results from the principal component analysis (PCA).

Climate variable	Botanical garden									*Loadings PCA1*	*Loadings PCA2*
	FRA	MAI	HAL	POT	BS	JEN	BN	MUE	OSN		
Sum of the monthly mean temperature in summer 2009 (June-August) in °C	57.9	56.6	54.4	54.4	53.6	53.2	53.5	52.9	52.7	***0.422***	*−0.227*
Sum of the monthly mean temperature of the vegetation period 2008 (August-October) in °C	42.8	42.0	41.6	41.0	41.4	40.6	40.9	41.0	40.5	***0.412***	*−0.186*
Sum of the monthly mean temperature of the vegetation period 2008 and 2009 in °C	147.0	144.4	138.5	138.2	137.1	135.5	136.8	136.1	135.2	***0.411***	*−0.258*
Sum of the monthly mean temperature of the vegetation period 2009 (April-September) in °C	104.2	102.4	96.9	97.2	95.7	94.9	95.9	95.1	94.7	*0.404*	*−0.269*
Precipitation in summer 2009 (June-August) in mm	205.5	163.2	151.1	152.1	139.2	173.3	182.9	209.3	198.1	*−0.140*	***−0.592***
Precipitation of the vegetation period 2009 (April-September) in mm	334.6	281.6	302.2	278.0	240.4	362.8	368.0	332.0	316.0	*−0.149*	***−0.399***
Minimum of monthly mean temperature 2008–2009	−1.9	−1.8	−2.1	−1.9	−0.7	−2.6	−0.8	−0.5	−0.2	*−0.279*	*−0.097*
Precipitation of the vegetation period 2008 (August–October) in mm	168.9	169.1	152.0	178.4	169.9	159.3	171.7	223.7	280.2	*−0.316*	*−0.244*
Precipitation of the vegetation period 2008 and 2009 in mm	503.5	450.7	454.2	456.4	410.3	522.1	539.7	555.7	596.2	*−0.317*	***−0.443***
*First axis scores of the PCA ( = PCA1)*	*3.32*	*2.76*	*0.99*	*0.45*	*0.32*	*−0.98*	*−1.35*	*−2.20*	*−3.30*		
*Second axis scores of the PCA ( = PCA2)*	*−2.66*	*−0.13*	*1.25*	*1.41*	*2.50*	*0.42*	*−0.54*	*−1.11*	*−1.13*		

Climate variables are arranged according to their loadings on the first PCA axis. Botanical gardens included in this study are sorted according to their scores of the first PCA axis. Scores and loadings of the Principal Component Analysis (PCA) are shown in italics. The three variables with highest absolute loadings per PCA axis are shown in bold letters. Botanical garden abbreviation: BN – Bonn, BS – Braunschweig, FRA – Frankfurt am Main, HAL – Halle (Saale), JEN – Jena Isserstedt, MAI – Mainz, MUE – Münster, OSN – Osnabrück, POT – Potsdam.

For the study period (June 2008–October 2009), monthly mean data for temperature and precipitation were obtained from the nearest official meteorological stations. Table 1 shows mean temperatures as well as accumulated temperature and precipitation sums at the nine study sites for the investigation period. To analyze the weather conditions during the study period, the climate data of all nine Botanical Gardens were subjected to a principal component analysis (PCA, prcomp procedure; R 2.15.2, R Development Core Team 2012). In the PCA, the Botanical Gardens were ordered along a clear gradient related to temperature on the first, and precipitation variables on the second axis (explaining 53% and 28% of the overall variance in climate conditions, respectively). Summer temperature of 2009 had the highest loading and was positively correlated with the first PCA axis scores (Table 1). The remaining temperature variables for the vegetation period were also highly positively correlated with the first PCA axis while precipitation variables and minimum temperature were negatively correlated with this axis (Table 1). Regarding the second PCA axis, summer precipitation in 2009 had the highest loading and was negatively correlated with the respective axis scores (Table 1). The Botanical Gardens were arranged from Osnabrück (OSN) to Frankfurt (FRA) and from Frankfurt (FRA) to Braunschweig (BS) along PCA axis 1 and 2, respectively (Table 1).

### Transplants and measurements

Fourteen herbaceous species were included in the experiment, two from each of seven genera. The two congeneric species are of similar growth form, have similar habitat preferences (Table S1), but differ in their geographical distribution range, in particular with respect to the longitudinal positions of their western range boundary (Figure S1). The following species were included in this transplant experiment (taxonomy according Jäger & Werner [27], plant range type as follows: oceanic-continental): *Carlina vulgaris* - *Ca*. *biebersteinii*, *Centaurea scabiosa* - *Ce*. *stoebe*, *Dianthus deltoides* - *Di*. *carthusianorum*, *Inula conyzae* - *I*. *hirta*, *Koeleria pyramidata* - *K*. *macrantha*, *Scabiosa columbaria* -*Sc*. *ochroleuca* and *Silene nutans* - *Si*. *otites*. The species are perennial plants which are all native to Europe (see distribution maps in Figure S1) and occur mainly in dry to semidry open grasslands [27]. All species had already been investigated in other glasshouse [21], [22] and field experiments [28].

Seeds of all species were collected in summer 2007 in Central Germany, using large populations to avoid negative effects of low genetic diversity (for geographical coordinates of the sampling localities see Table S1). Seedlings of all species were raised under controlled standardized glasshouse conditions in spring 2008. In June 2008, the young seedlings were transplanted into the plots. To ensure initial establishment, plants were watered regularly for one month. There were some species for which not enough seedlings were available, resulting in three species pairs that could not be planted in all gardens. These missing species pairs were randomly assigned to all gardens, except for those at the ends of the climate gradient which received all seven genera. We made sure that no more than one species pair was missing in any of the nine gardens.

At the beginning of the experiment, the leaf number of every transplant was counted to calculate relative growth rates according to Hunt [29]. At the end of the experiment, in October 2009, survival, flowering status, number of leaves, number of flowering units, plant height, specific leaf area (SLA) and the proportion of consumed leaf area (visually assessed) were recorded. Additionally, aboveground biomass was harvested and weighed after drying at 70°C for 48 hours.

All data are available at http://data.idiv.de/repo/data_Welk_etal_PlosOne.xls.

### Experimental setup

In every garden one study plot consisting of 16 subplots of one by one metre area each was established (Figure S2). Each subplot was divided in four rows and four columns, resulting in 16 planting positions (Figure S2). One individual per plant species was planted in every subplot at randomly chosen positions resulting in a maximum of 14 transplants (and two empty positions) per subplot. For the competition treatment, seeds of *Festuca rubra* (cultivar *Wilma*, RUDLOFF Feldsaaten GmbH, Bad Schwartau, Germany) were sown (5 g/m^2^) on eight subplots at the time of planting of the transplants (Figure S2). All subplots were regularly weeded except for *Festuca rubra* in the competition subplots. For the mollusc exclosure we regularly applied a mollusc repellent (Ferramol, W. Neudorff GmbH KG, Emmerthal) on eight of the subplots. In spring 2009, these plots were additionally equipped with metal frame fences to exclude molluscs (IRKA, R+M Gartenbedarf, Rehling, www.der-schneckenzaun.de). The competition and mollusc exclosure treatments were fully crossed and all treatment combinations were replicated four times per Botanical Garden (Figure S2), resulting in a total of 144 subplots and 1824 transplant individuals.

### Statistical analyses

All response variables (survival, incidence of flowering, RGR of leaf number, aboveground biomass, plant height, number of flowering units, SLA and proportion of consumed leaf area biomass) were analysed with separate generalized linear mixed effect models (GLMM, proc glimmix, type III SS, SAS 9.2, SAS Institute Inc. 2008). For survival and incidence of flowering, a logit-link function and binomial error distribution were used, while the GLMM for all other response variables had an identity-link function and Gaussian error distribution. To identify the most relevant climatic drivers of the different response variables we included the scores of the first and second PCA axes as covariates in the models. Fixed categorical factors in all models were plant range type (oceanic, continental), competition (presence/absence) and herbivory (presence/absence). All possible two and three-way interactions of fixed factors and covariates were included. Subplot identity (nested in the interaction of garden identity, herbivory and competition treatment) and species identity (nested in plant range type) entered the models as crossed random factors.

Unbiased least square means (LS means) and standard errors were calculated using the LSMEANS statements in SAS 9.2 (SAS Institute Inc. 2008) and used to produce graphs. Tukey post-hoc tests were calculated for contrasts between treatment combinations. All graphs were produced with R 2.15.2 (R Development Core Team 2012).

## Results

### Main effects of climate, competition and herbivory

The climate gradient significantly affected survival, aboveground biomass, plant height, number of flowering units, SLA and proportion of leaf area consumed (Table 2). While temperature, which was captured by the first PCA axis, had no significant effect on any response variable, precipitation, with high loadings on the second PCA axis, played a major role. Survival (p = 0.005), plant height (p = 0.013) and number of flowering units (p<0.001) decreased with decreasing precipitation (i.e. increasing PCA2 scores), while proportion of consumed leaf area increased (p = 0.020). Additionally, there were significant interactions of both PCA axes for survival (p = 0.002), aboveground biomass (p = 0.005), plant height (p = 0.038), number of flowering units (p<0.001) and specific leaf area (p = 0.042, Table 2).

**Table 2:** The effect of climatic covariates (PCA axes 1 and 2, representing temperature and precipitation effects, respectively), competition, herbivory and plant range type on the different variables of plant performance measured in the Botanical Gardens.

Effect		Survival rates			Incidence of flowering			RGR of leaf number			Aboveground biomass			Plant height			Number of flowering units			Specific leaf area (SLA)			Proportion of consumed leaf area		
	DF_num_	DF_den_	F		DF_den_	F		DF_den_	F		DF_den_	F		DF_den_	F		DF_den_	F		DF_den_	F		DF_den_	F	
PCA1	1	1638	0		1500	2.78		1243	1.04		1225	0.13		1449	0.46		1499	2.5		1172	0.5		1237	0.16	
PCA2	1	**1638**	**7.84**	**	1500	0.15		1243	2.03		1225	2.04		**1449**	**6.20**	*	**1499**	**16.72**	***	1172	1.01		**1237**	**5.40**	*
PCA1×PCA2	1	**1638**	**10.06**	**	1500	1.41		1243	1.23		**1225**	**8.08**	**	**1449**	**4.30**	*	**1499**	**19.61**	***	**1172**	**4.13**	*	1237	0.26	
Competition (C)	1	129	0.28		**130**	**13.52**	***	**130**	**16.26**	***	**128**	**53.13**	***	130	3.02		**130**	**28.58**	***	**130**	**5.37**	*	**130**	**18.96**	***
C×PCA1	1	1638	0.25		1500	1.15		1243	0.01		1225	0		1449	0.65		**1499**	**4.12**	*	1172	0.07		1237	0.48	
C×PCA2	1	1638	2.21		1500	0.23		1243	0.47		1225	1.19		1449	0.04		1499	0.23		1172	0.60		1237	0.35	
C×PCA1×PCA2	1	1638	0.32		1500	1.37		1243	1.17		1225	0.77		1449	0.15		**1499**	**7.76**	**	1172	0.67		1237	0.88	
Herbivory (H)	1	129	0.87		130	1.72		130	0.66		128	0.04		**130**	**4.78**	*	130	0.44		130	2.41		**130**	**14.40**	***
H×PCA1	1	1638	0		1500	2.11		1243	0.09		1225	0.01		1449	0.68		1499	0.34		1172	0.11		1237	0	
H×PCA2	1	1638	0.26		**1500**	**4.90**	*	1243	0.39		1225	0.03		1449	0.72		1499	1.38		1172	0		1237	1.66	
H×PCA1×PCA2	1	1638	0.01		1500	0.47		1243	0.50		1225	0		1449	0.46		1499	1.29		1172	0.02		1237	0.38	
C×H	1	129	0.08		130	0.25		130	0.64		128	0.19		130	0.06		130	2.32		130	0.20		**130**	**8.47**	**
C×H×PCA1	1	1638	1.63		1500	0.17		1243	0.76		1225	0.07		1449	0.01		1499	0		1172	0.07		1237	0.05	
C×H×PCA2	1	1638	0.35		1500	2.47		1243	1.15		1225	1.11		1449	0.30		1499	0.17		1172	0.48		1237	2.74	
Range type (R)	1	12	1.49		12	0.50		12	2.50		12	0.02		12	0.07		12	0.63		12	1.13		12	0.37	
R×PCA1	1	1638	0.06		1500	1.67		1243	0.80		1225	0.02		1449	2.01		1499	0.80		1172	0.03		1237	0.45	
R×PCA2	1	1638	0.56		**1500**	**5.64**	*	1243	0		1225	0.54		**1449**	**6.65**	*	1499	0.51		1172	0.86		1237	0.88	
R×PCA1×PCA2	1	1638	2.29		1500	0.78		1243	0.40		1225	0.39		1449	0.53		1499	3.19		1172	2.42		1237	0.03	
C×R	1	**1638**	**5.07**	*	**1500**	**4.71**	*	1243	1.09		**1225**	**6.97**	**	1449	0.01		**1499**	**5.74**	*	1172	0.07		1237	1.59	
C×R×PCA1	1	1638	2.23		1500	0.01		1243	0.23		1225	0.44		1449	3.42		1499	0.62		1172	0.48		1237	0.11	
C×R×PCA2	1	1638	0.84		**1500**	**3.96**	*	1243	1.38		**1225**	**4.57**	*	1449	1.51		1499	3.20		1172	2.64		1237	1.27	
H×R	1	1638	0.44		**1500**	**7.76**	**	1243	0.03		1225	0.95		1449	1.26		1499	0.08		1172	0.02		**1237**	**4.91**	*
H×R×PCA1	1	1638	0.05		1500	2.36		1243	0.12		1225	0.50		1449	0.50		1499	1.22		1172	0.13		1237	0.01	
H×R×PCA2	1	1638	0.18		1500	0.02		1243	0.02		1225	0.01		1449	0.82		1499	0.99		1172	0.06		1237	0.03	
R×C×H	1	**1638**	**4.85**	*	1500	2.94		1243	3.31		1225	0.20		**1449**	**5.18**	*	**1499**	**5.29**	*	1172	0.50		1237	0.12	

The table shows degrees of freedom for the numerator and denominator (DF_num_ and DF_den_, respectively as well as F values from a type III Anova based on generalized linear models. Bold F values indicate significant effects. ***p<0.001, **p<0.01, *p<0.05.

The competition and mollusc exclosure treatments had strong effects on all response variables except survival (Table 2). This demonstrates that our experimental subplot manipulation of biotic interactions was effective and that these two biotic factors were key determinants of plant performance across all species. Competition significantly reduced the incidence of flowering (by −7.1%, referring to LSmeans estimates from the GLMM, p<0.001), RGR of leaf number (by −59.2%, p<0.001), aboveground biomass (by −40.8%, p<0.001) and number of flowering units (by −35.5%, p<0.001). Furthermore, there was an increase in SLA (by +6.2%, p = 0.022) and proportion of consumed leaf area (by +50.1%, p<0.001) when competitors were present. Mollusc exclosure resulted in increased plant height (by +3.5%, p = 0.031) and lower proportions of consumed leaf area (by −29.7%, p<0.001). Additionally, proportions of consumed leaf area showed a significant interaction effect of competition with herbivory (p = 0.004): While the mollusc exclosures reduced the amount of consumed leaf area by −10.3% in absence of competition, the reduction was −40.5% in presence of competition.

### Interaction of climate with impacts of competition and herbivory

In general, the climate gradient had no effects on the outcome of the biotic subplot manipulations for most response variables (Table 2). Along the first PCA axis (temperature) the competition effect on number of flowering units changed (p = 0.043, Table 2). With increasing temperature (i.e. increasing PCA1 scores), the number of flowering units increased when competitors were absent, while the number of flowering units remained unchanged when competitors were present. The effect of mollusc exclosure changed along the second PCA axis (precipitation) for incidence of flowering (p = 0.027, Table 2). With decreasing precipitation (i.e. increasing PCA2 scores) flowering was observed less frequently when molluscs were excluded, while flowering increased in frequency in the presence of mollusc herbivory.

### Effects of plant range type and the interactions of plant range type with climate, competition and herbivory

No significant main effects of plant range type were observed for any of the response variables (Table 2). This also applied to SLA, showing that the two representatives for plant range types did not differ in basic functional traits. However, significant interactions indicated that species of different plant range types responded differently along the climate gradient and to the subplot treatments (Table 2).

**Figure 2:**
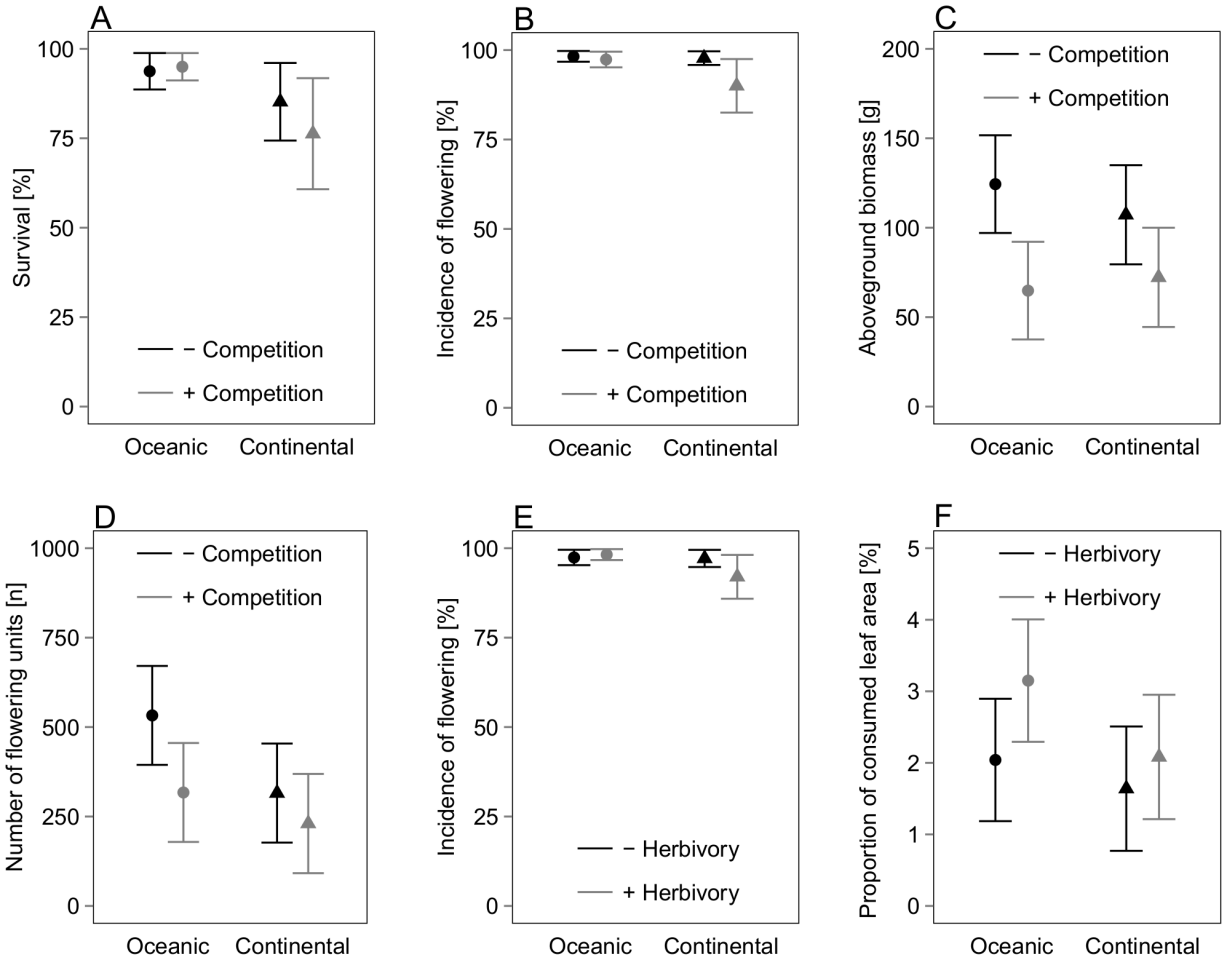
Significant interactive effects of plant range type with competition on A) survival, B) incidence of flowering, C) aboveground biomass and D) number of flowering units, and interactive effects of plant range type with herbivory on E) incidence of flowering and F) proportion of consumed leaf area. Graphs are plotted with LSmeans estimates and standard errors derived from the GLMM's. For statistical details see Table 2.

Survival, incidence of flowering, aboveground biomass and number of flowering units were significantly affected by the interaction of plant range type with competition (Table 2). The plants of the oceanic range type survived slightly better in presence of competitors (+1.6%), whereas those of the continental range type had clearly lower survival rates (−9.0%) when competitors were present (p = 0.025, Fig. 2A). Similarly, plants with continental range type flowered less frequently in presence of competitors (−7.7%), whereas flowering of oceanic plants remained nearly unaffected by competition (−0.9%, p = 0.030, Fig. 2B). In contrast, aboveground biomass (oceanic −47.9%, continental −32.6%, p = 0.008) and number of flowering units (oceanic −40.5%, continental −27.0%, p = 0.017) were generally negatively affected by competition, with stronger negative effects on plants with oceanic than on continental range type (Fig. 2C,D).

With respect to the herbivory treatment, plant range type was important for the incidence of flowering (p = 0.007) and proportion of consumed leaf area (p = 0.027, Table 2). Plants of the continental range type showed a decrease in the proportion of flowering individuals (−4.8%) in the presence of molluscs while those of the oceanic range type displayed a slight increase (+0.6%, Fig. 2E). Without mollusc exclosure, plants of the oceanic range type were consumed more frequently than plants of the continental range type (Fig. 2F).

**Figure 3:**
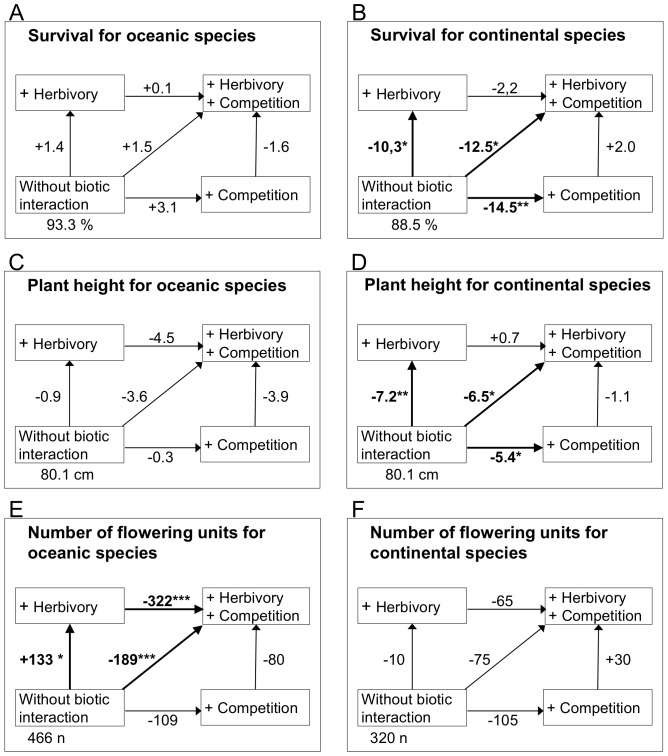
Summary of interactive effects of competition and herbivory on species with different plant range types for survival (A,B), plant height (C,D) and number of flowering units (E,F). Effects for the oceanic species are illustrated on the left side (A,C,E), for continental on the right side (B,D,F). The values in the bottom left corner are LSmeans estimates calculated from the GLMM for the treatment without biotic interactions. The values on the arrows are differences in the LSmeans estimates of this treatment-combination indicating the direction and strength of the relation. Units are percentage (A,B), cm (C,D) and numbers (E,F). Bold arrows indicate significant effects according to the Tukey post hoc-test. ***p<0.001, **p<0.01, *p<0.05.

There were also threefold significant interactions of competition, herbivory and plant range type. The plant range types displayed contrasting response patterns to herbivory and competition in survival (p = 0.028, Fig. 3A,B), plant height (p = 0.023, Fig. 3C,D) and number of flowering units (p = 0.022, Fig. 3E,F, Table 2). Survival and plant height of continental plants were strongly negatively affected by the single effects of herbivory and competition (Fig. 3B,D). These effects had the same magnitude and were not additive when mollusc herbivory occurred in combination with competition. Survival and plant height of oceanic plants remained unaffected by the biotic treatments (Fig. 3A,C). The contrasting pattern was observed for the number of flowering units, where oceanic plants were strongly affected by biotic interactions (Fig. 3E), while continental plants were not (Fig. 3F). In oceanic plants, herbivory and competition had opposing effects on number of flowering units. While herbivory alone caused a significant increase in number of flowering units, competition alone had no significant effect. However, when competitors were present in addition to herbivores, the number of flowering units of oceanic plants strongly decreased (Fig. 3E). Neither RGR of leaf number nor the proportion of consumed leaf area showed significantly different effects for the contrasting range types in the presence or absence of herbivores or competition (Table 2).

**Figure 4:**
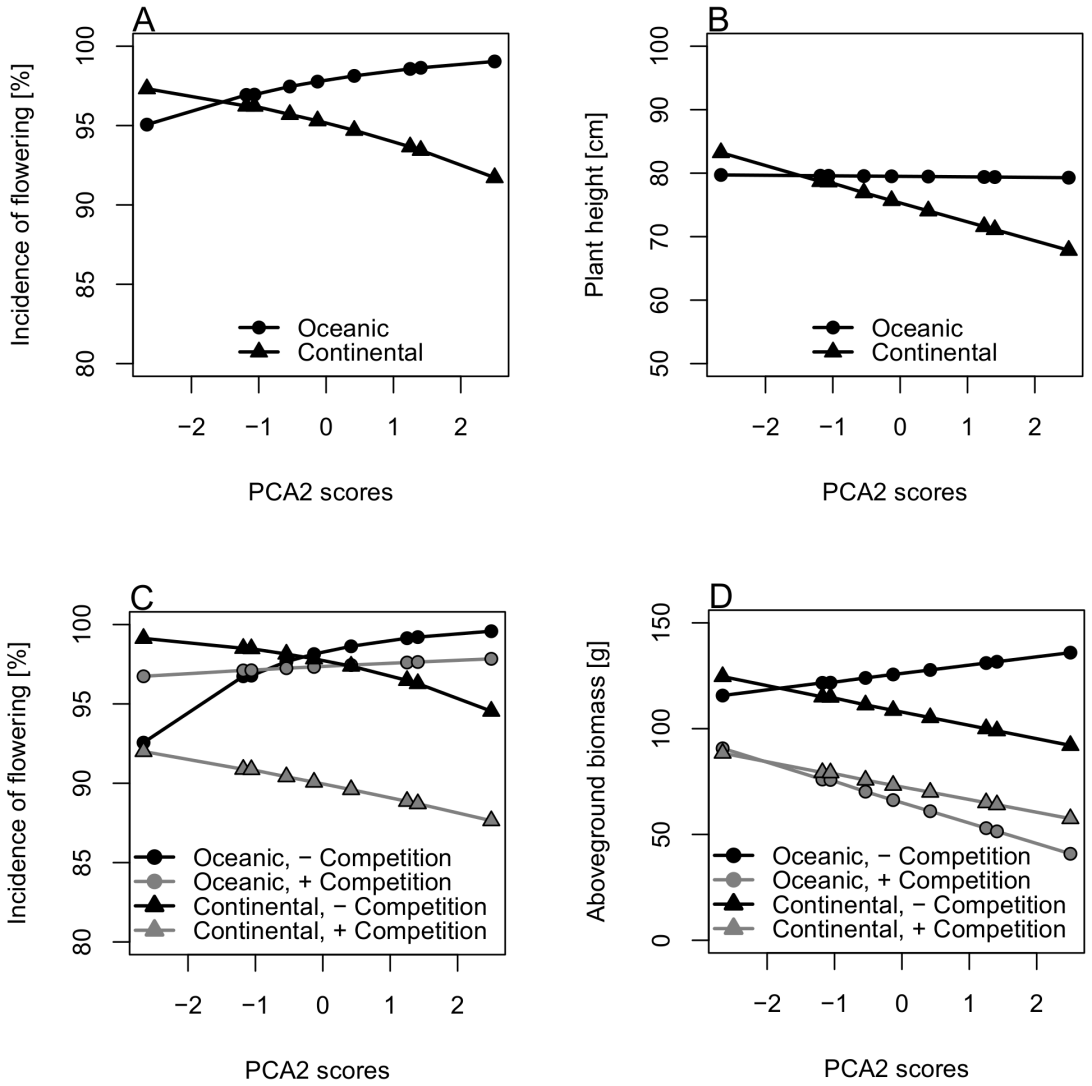
Significant effects of the climate gradient (climate covariate) on plants with different plant range types for A) incidence of flowering and B) plant height, and in interaction with competition for C) incidence of flowering and D) aboveground biomass. Climate covariates are PCA 2 scores which are negatively correlated with precipitation sums of the vegetation period during the experiment (Table 1). Graphs are plotted with LSmeans estimates derived from the GLMM's. For statistical details see Table 2.

The responses to the climatic variables of plants of contrasting range types differed for incidence of flowering (PCA2, p = 0.018, Fig. 4A) and plant height (PCA2, p = 0.010, Fig. 4B, Table 2). Incidence of flowering of continental plants decreased with decreasing precipitation (i.e. increasing PCA2 scores) while that of oceanic plants increased (Fig. 4A). Similarly, plant height of continental plants decreased with decreasing precipitation) but remained constant in oceanic plant species (Fig. 4B). At the moist end of the gradient, plants of the continental range type flowered more frequently and were taller than oceanic ones, while on the dry end the pattern was reversed (Fig. 4A,B).

Furthermore, along the precipitation gradient, the effect of competition differed between range types for incidence of flowering (PCA2, p = 0.047, Fig. 4C) and aboveground biomass (PCA2, p = 0.033, Fig. 4D, Table 2). For both response variables, oceanic plants showed changed climate responses when competition was manipulated. Incidence of flowering and aboveground biomass strongly increased with decreasing precipitation (i.e. increasing PCA2 scores) when competitors were absent (Fig. 4C). In the presence of competition, oceanic plants showed only a slight increase in incidence of flowering (Fig. 4C) and a decrease in aboveground biomass with decreasing precipitation (i.e. increasing PCA2 scores, Fig. 4D). In contrast, plants with continental plant range type showed almost no changes in their climate responses when competition was manipulated. With decreasing precipitation (i.e. increasing PCA2 scores) incidence of flowering and aboveground biomass of continental plants decreased, both in the absence and presence of competitors (Fig. 4C,D).

## Discussion

The climate conditions in our transplant experiment were characterized by a distinct gradient from oceanic to continental climate. Across all species included in our study, temperature was less important for performance variation than precipitation. Plants benefited from higher precipitation in terms of higher survival, taller growth and increasing number of flowering units. Additionally, plant growth and reproduction were strongly negatively affected by competition and partly by mollusc herbivory, indicating that competition was more detrimental than herbivory. Biotic interactions affected all response variables except plant survival. This indicates that the relative importance of biotic interactions and climate differed among the different response variables.

### Effects of climate on biotic interactions

In our first hypothesis, we expected that the negative effect of competition and herbivory is mitigated under the more stressful continental climate conditions. This was not the case for survival and plant growth. Biotic interactions only changed the response along the climate gradient in the case of variables related to flowering. Competition generally reduced aboveground biomass and growth, irrespective of climate. Restrictions in water supply at the dry end of the climatic gradient did not aggravate the effect of competition, probably because target plants and competitors were affected likewise. However, we also have to consider that the fertile garden soils used in our experiment have affected the interaction of climate and competition. While competition is expected to become more pronounced under fertile conditions, drought effects might have been reduced. In contrast to competition, mollusc herbivory had no impact on biomass production and was independent of the climate conditions in our experiment, except for incidence of flowering. This does not confirm the results of a recent global meta-analysis of Rodríguez-Castañeda [30], who found that the effect of general herbivory on plant performance increased in moister ecosystems. Bruelheide & Scheidel [11] described increased slug herbivory with decreasing altitude, which was reflected in increasing temperatures, and overall, more benign growth conditions. A potential explanation for this mismatch to our results might be that the climate gradient used in our study was not steep enough to evoke climate-dependent herbivory effects on plant performance. Additionally, the generally high slug abundances in Botanical gardens might have uncoupled the climate-herbivory relationship, which emphasises the need for assessing mollusc densities in future studies. Given the large distance between the experimental sites, we were not able to count molluscs during rain events or to quantify mollusc activity [11], [16].

### Interaction of climate with plant range type

We secondly hypothesised that the changing climate conditions in our study have a different impact on the transplants with respect to their range type. The expectation that species of the oceanic plant range type perform better at the oceanic end of the climate gradient and those of the continental plant range type at the continental end of the climate gradient was not confirmed. Instead, we even found opposing patterns with increased incidence of flowering, plant height and aboveground biomass for continental plants under moister conditions and for oceanic plants under drier conditions. These results support the outcome of previous experiments in the field and the greenhouse, where analyses with the same species set revealed similar responses to manipulated climate change [28] or altered soil moisture conditions [21], [22]. Nevertheless, at least for the continental species, we expected a poorer performance in the more oceanic climate since the experimental setup covered an area extending beyond their western distribution limit (Figure S1). However, the general impression was that plants of the continental range type responded stronger to the climatic gradient than those of the oceanic range type, especially with plant height. In contrast to our findings, numerous transplant experiments have described a decreased fitness beyond a species' current range [31]. However, most of these species' range boundaries were studied along latitudinal or altitudinal climate gradients [32], [33], where temperature is the dominant driver. In contrast, the climate gradient covered in our experiment is less simple as higher temperatures that favour growth rates are counteracted by increasing drought risk. Consistent with our results, Stanton-Geddes et al. [34] observed fitness declines towards the northern range edge but not to the western range edge of *Chamaecrista fasciculata*, a widespread annual legume from North America. However, we also have to consider that short-term experiments, as presented here, might perhaps not be able to detect clear home-site advantages. For example, in a transplant experiment monitored over 30 years Bennington [35] encountered increasingly stronger home-site advantages for ecotypes of two arctic plant species with time. Our study design also did not allow for assessing the impact of climate on population demography as we did not focus on recruitment. Given that our species are almost all long-lived, although some of them are hapaxanth (Table S1), a 2 years-study period is certainly not sufficient to estimate the impact of climate on long-term survival and demography. This clearly underlines the need for long-term studies.

### Interaction of competition and herbivory with plant range type

When climatic factors failed to explain species range limitation, biotic interactions have often been made accountable for directly limiting distribution ranges [31], [36]. For example, Engels & Jensen [37] found that plant species from salt marshes performed similarly well in both freshwater and saltwater marshes, when competition was excluded. Similarly, in our experiment, plants with continental range type survived much better in absence of competition than those with oceanic plant range type. In addition, continental plants flowered more frequently when competitors or herbivores were removed or excluded, respectively. This sensitivity to competition and herbivory of continental plants was not modified by climate. This indicates that the geographic distribution of continental plants is not directly limited by climate conditions but by other factors, such as competition. However, biotic interactions might act on different spatial scales. In a tundra study system Le Roux et al. [38] found that small scale species distribution was shaped by horizontal biotic interactions (i.e. competition) rather than by vertical interactions (i.e. herbivory). Similarly, our experimental evidence on survival and incidence of flowering supports our third hypothesis stating that continental plants are more vulnerable to competition and herbivory than oceanic plants. However, oceanic plants were stronger negatively affected by competition in biomass and flower production than continental plants. Regarding these inconsistent results, the third hypothesis that continental plants are more susceptible to competition or herbivory has to be rejected.

Although individuals of both plant range types suffered from biotic stress, they showed different strategies to cope with that. Particularly, the combined effect of competition and herbivory caused different plant responses with respect to plant range type. Only plants of continental range type displayed lower survival rates and smaller plant height in presence of competitors and herbivores. In contrast, oceanic plants were significantly affected in their flower production by herbivory alone and in combination with competition. The reduced flower production of the oceanic plants might be an effect of the compensatory response to herbivory and demonstrates a shift in resource allocation to enhanced plant growth [39]. Further reproductive traits such as number of seeds, seed mass and seed dispersal mode, have also profound effects on plant persistence [40] but could not be assessed in our experiment. Nevertheless, survival of plant individuals is ecologically essential to maintain a population. A synthesis of all our results indicates, that continental plants run a higher risk to suffer from competition and herbivory than oceanic plants, particularly if they are subjected to the combination of both competition and herbivory.

### Conclusion

From the results of our experiment we can conclude that, at the geographical scale of Germany, the continentality gradient is of minor direct importance for species of the considered plant range types compared to negative biotic interactions. Competition and herbivory affected particularly the performance of individuals of continental plant range type. There was only weak evidence for the assumption that the impact of competition and herbivory should vary with climate. In consequence, predictions of future geographic range dynamics of plants species have to be considered with caution, especially when based solely on occurrence data and climatic variables. Mechanistic models would be more suitable, but to devise such models a better understanding of plant ecology is needed [41]. Consequentially, Wisz et al [42] recommended targeted long-term field monitoring approaches. Such long-term field experiments should not only include species of different plant range types but also measure demographic rates, which requires the assessment of sexual and vegetative recruitment.

## Supporting Information

Figure S1 **Distribution maps of the congeneric species pairs used in the study.** A – *Carlina*, B – *Centaurea*, C – *Dianthus*, D – *Inula*, E – *Koeleria*, F – *Scabiosa*, G - *Silene*. Species which were assigned to oceanic range types are coloured in blue, continental are coloured in red. Violet colour indicates range overlap of the two species The Botanical Gardens where the experimental sites were located are shown as black dots. Details on the compilation of the data for these distributions maps are given in Hofmann et al. (2013).
(DOCX)

Figure S2 **Plot scheme of the experimental design in every Botanical Garden.** All treatments were randomly assigned to subplots and plants were randomly assigned to planting positions. All species were planted into subplots. Symbols: - C  =  absence of competitors (regular weeding), + C  =  presence of competitors (*Festuca rubra*), - H  =  slug herbivore exclusion (subplot with metal frame and slug repellents), + H  =  without slug herbivore exclusion (subplot without metal frame).
(DOCX)

Table S1 **Species characteristics (Jäger & Werner 2005) and coordinates of the localities where seeds were collected.** Growth form: eg = evergreen, sg = summergreen, hc =  hemicryptophyte, p =  perennial.
(DOCX)
