# Supplementary material for: Biotic Interactions Overrule Plant Responses to Climate, Depending on the Species' Biogeography
Source: PLoS One. 2014 Oct 30;9(10):e111023. doi: 10.1371/journal.pone.0111023 (PMC4214694; doi:10.1371/journal.pone.0111023)

**Figure S1.** Distribution maps of the congeneric species pairs used in the study: A – *Carlina*, B – *Centaurea*, C – *Dianthus*, D – *Inula*, E – *Koeleria*, F – *Scabiosa*, G - *Silene*. Species which were assigned to oceanic range types are coloured in blue, continental are coloured in red. Violet colour indicates range overlap of the two species The Botanical Gardens where the experimental sites were located are shown as black dots. Details on the compilation of the data for these distributions maps are given in Hofmann et al. (2013).


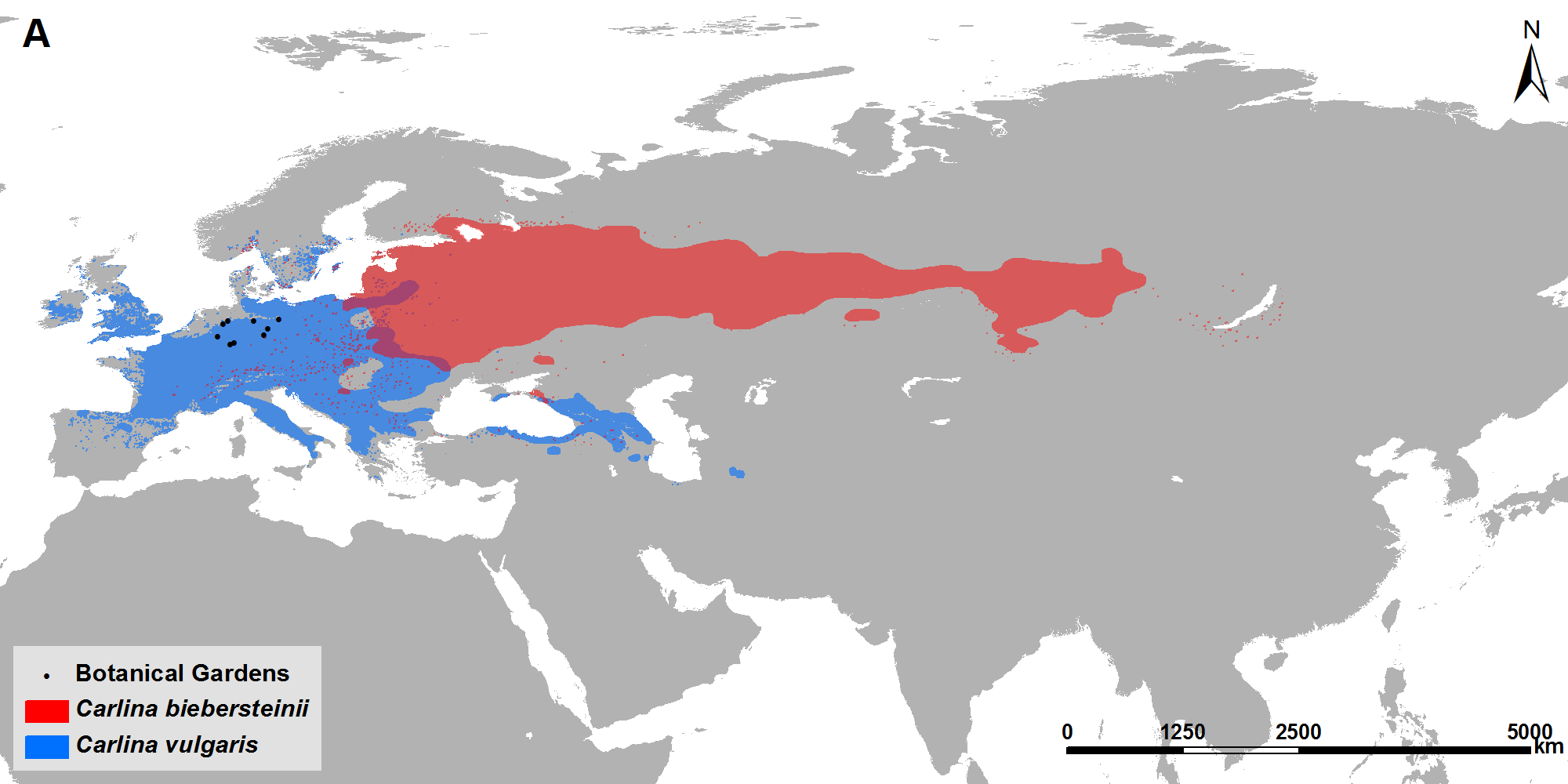


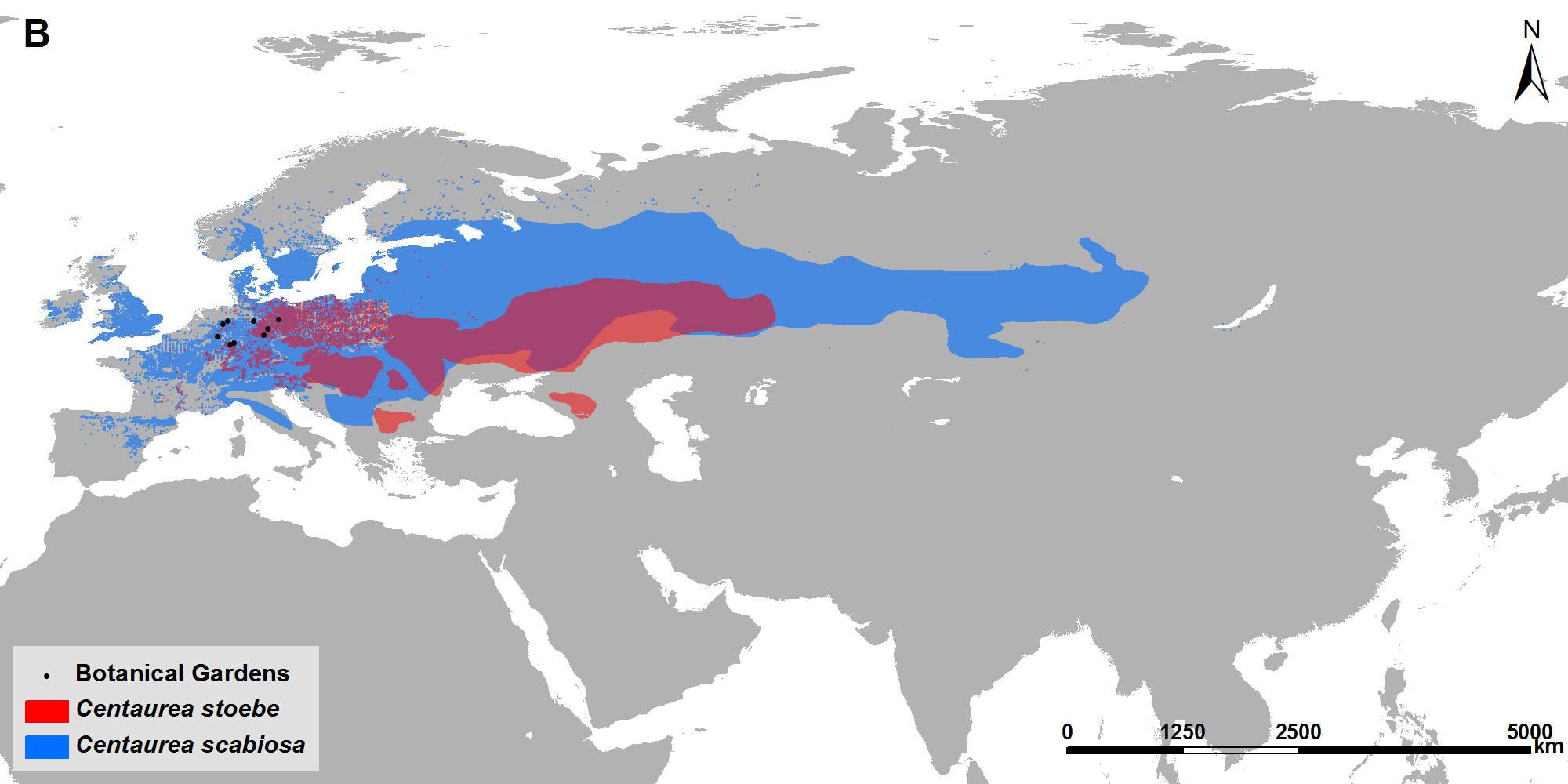


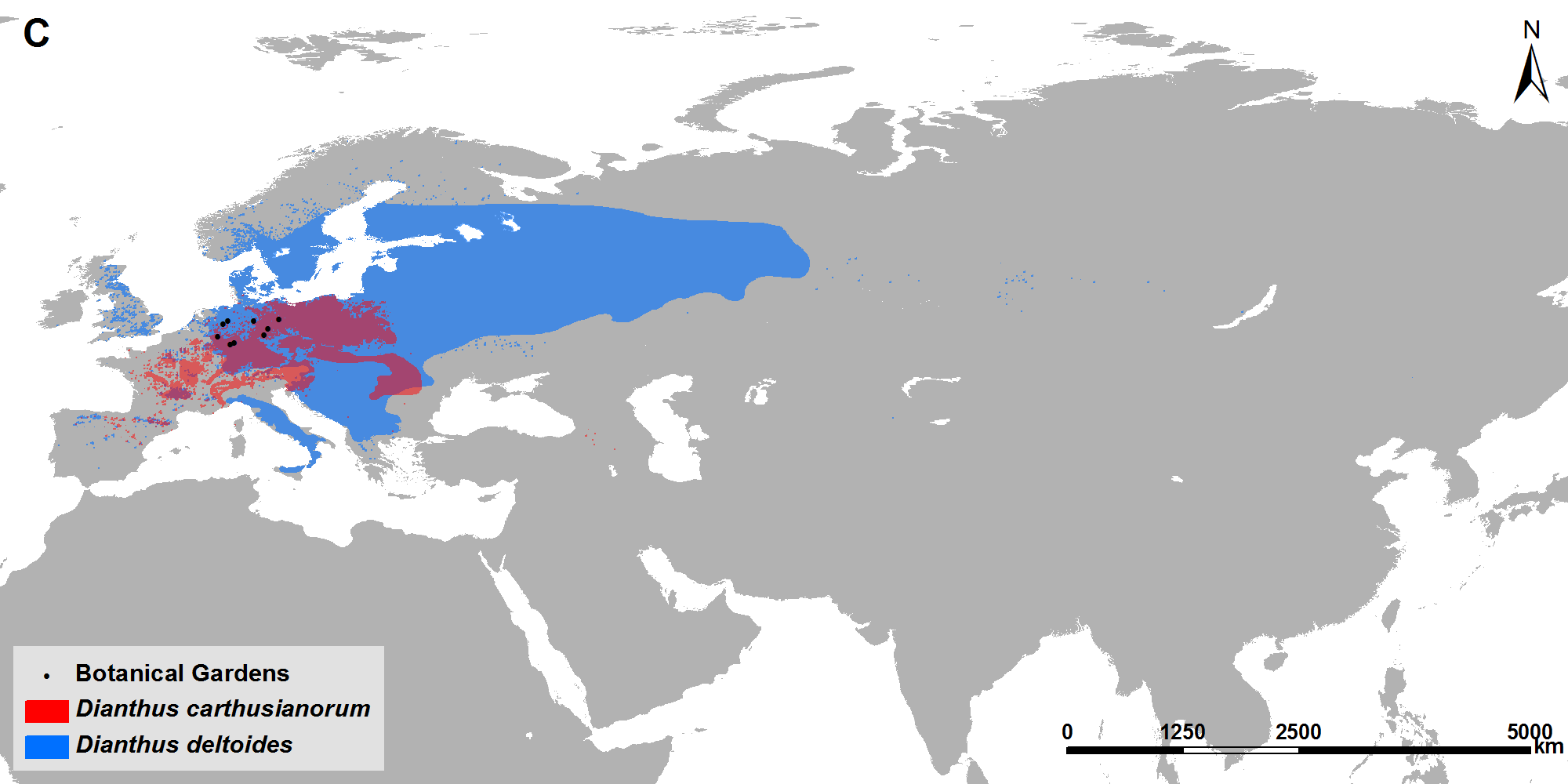


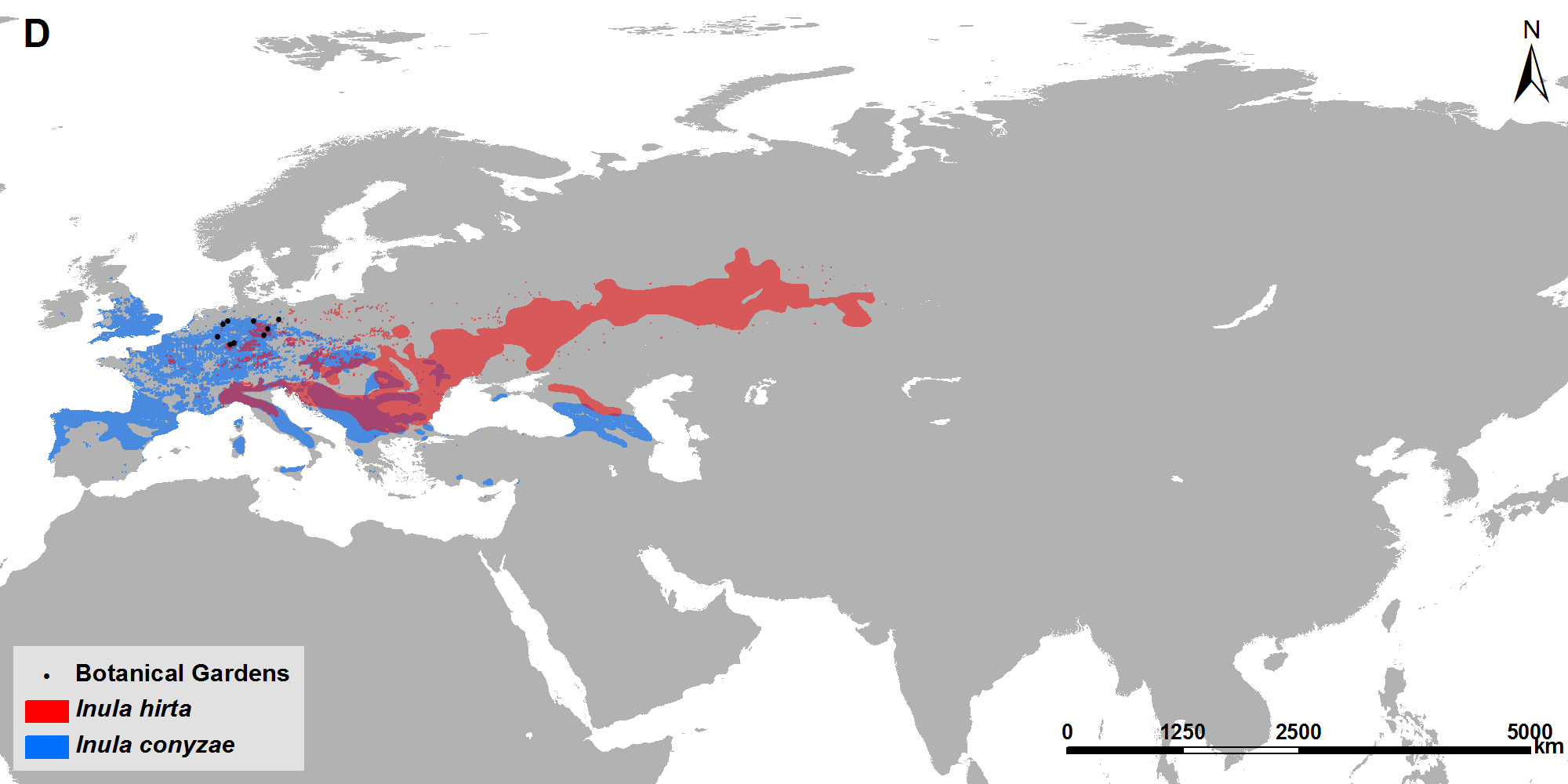


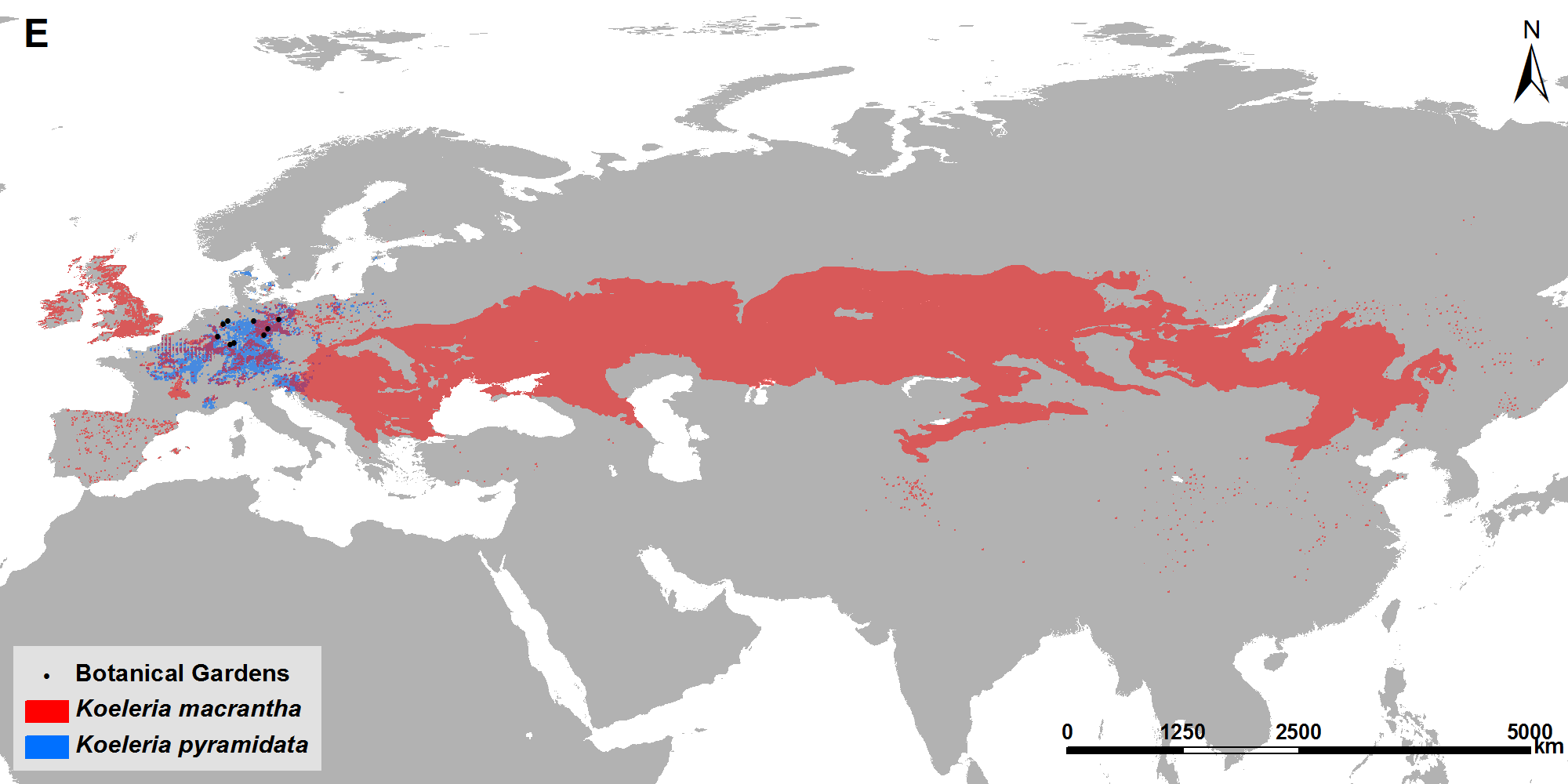


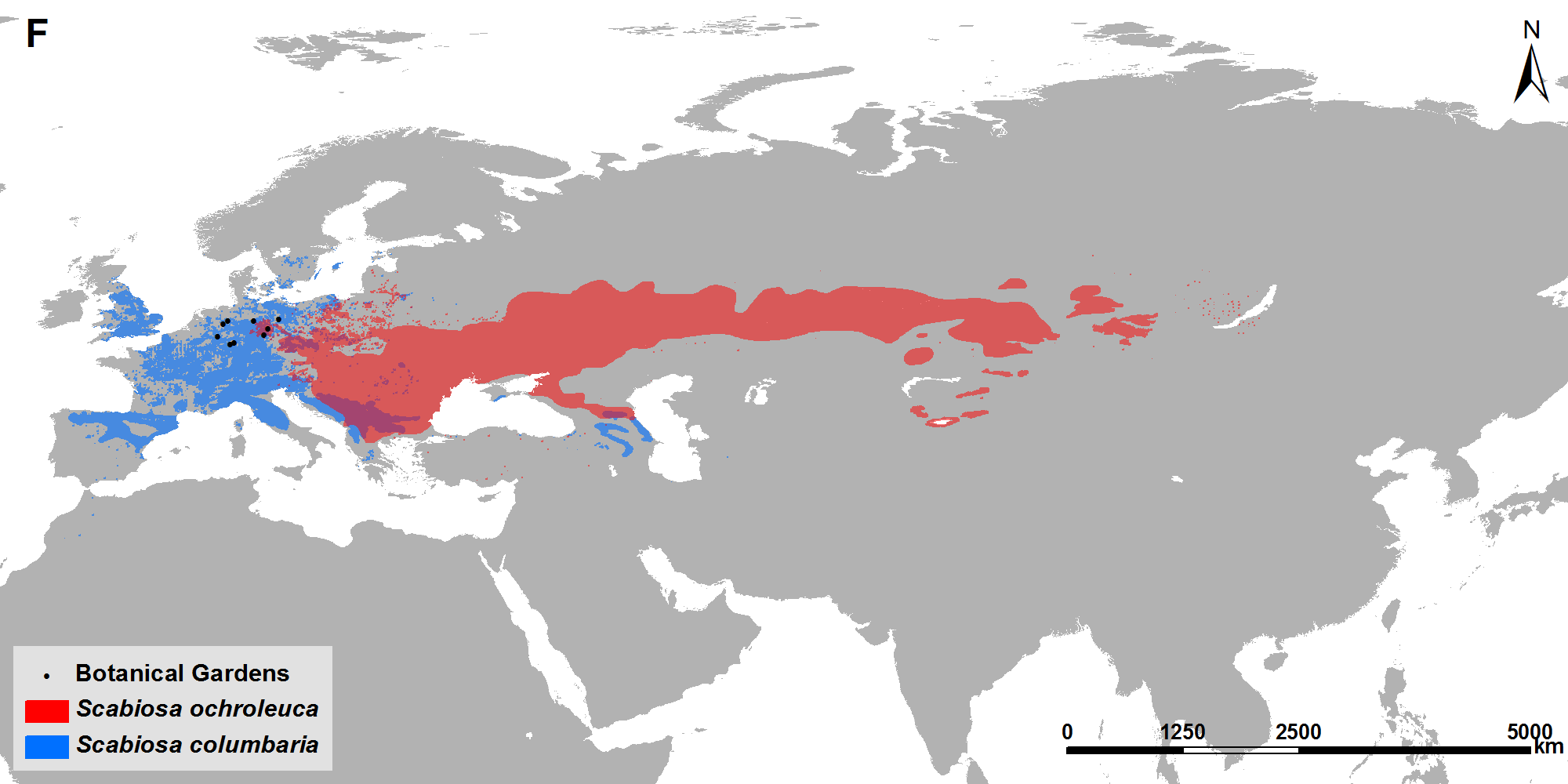


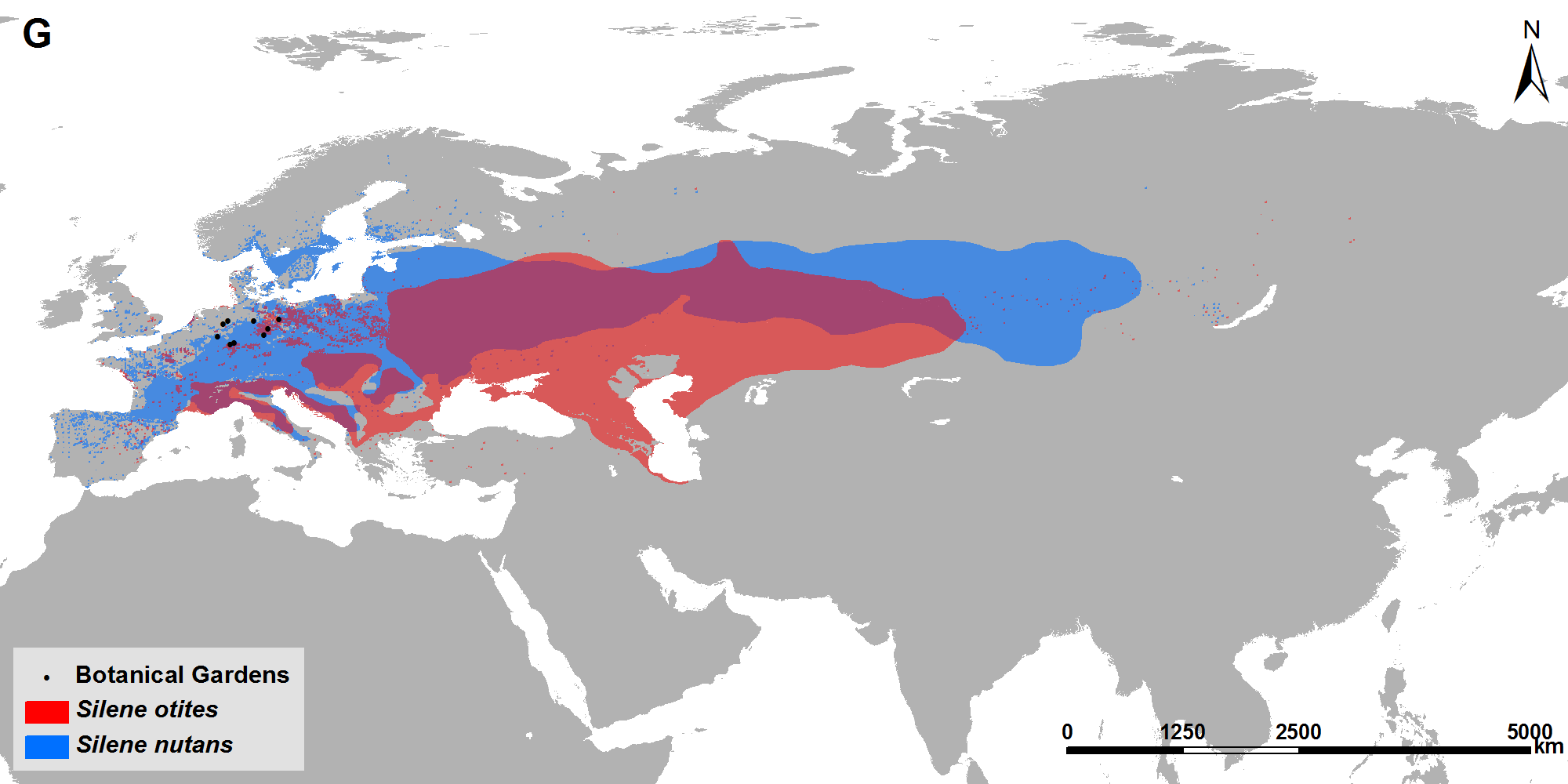

Supplement: Figure S1 — Distribution maps of the congeneric species pairs used in the study. A – Carlina, B – Centaurea, C – Dianthus, D – Inula, E – Koeleria, F – Scabiosa, G - Silene. Species which were assigned to oceanic range types are coloured in blue, continental are coloured in red. Violet colour indicates range overlap of the two species The Botanical Gardens where the experimental sites were located are shown as black dots. Details on the compilation of the data for these distributions maps are given in Hofmann et al. (2013). (DOCX) [file pone.0111023.s001.docx]
