# Supplementary material for: Biotic Interactions Overrule Plant Responses to Climate, Depending on the Species' Biogeography
Source: PLoS One. 2014 Oct 30;9(10):e111023. doi: 10.1371/journal.pone.0111023 (PMC4214694; doi:10.1371/journal.pone.0111023)
Supplement: Figure S2 — Plot scheme of the experimental design in every Botanical Garden. All treatments were randomly assigned to subplots and plants were randomly assigned to planting positions. All species were planted into subplots. Symbols: - C = absence of competitors (regular weeding), + C = presence of competitors (Festuca rubra), - H = slug herbivore exclusion (subplot with metal frame and slug repellents), + H = without slug herbivore exclusion (subplot without metal frame). (DOCX) [file pone.0111023.s002.docx]

**Figure S2.** Plot scheme of the experimental design in every Botanical Garden. All treatments were randomly assigned to subplots and plants were randomly assigned to planting positions. All species were planted into subplots. Symbols: - C = absence of competitors (regular weeding), + C = presence of competitors (*Festuca rubra*), - H = slug herbivore exclusion (subplot with metal frame and slug repellents), + H = without slug herbivore exclusion (subplot without metal frame).

**
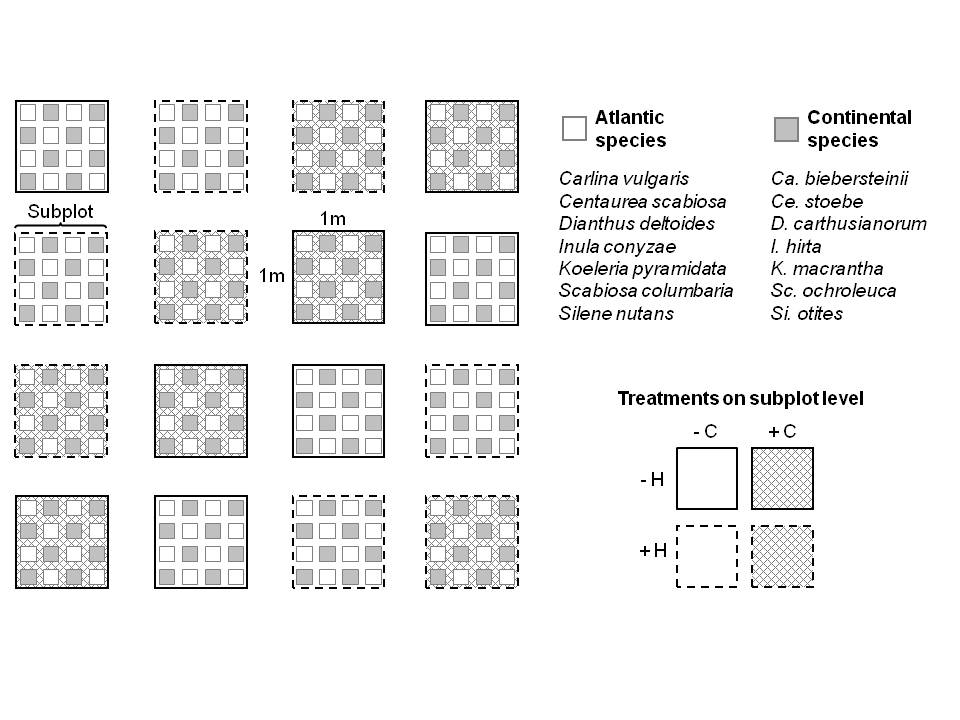
**
