# Supplementary material for: Biotic Interactions Overrule Plant Responses to Climate, Depending on the Species' Biogeography
Source: PLoS One. 2014 Oct 30;9(10):e111023. doi: 10.1371/journal.pone.0111023 (PMC4214694; doi:10.1371/journal.pone.0111023)
Supplement: Table S1 — Species characteristics (Jäger & Werner 2005) and coordinates of the localities where seeds were collected. Growth form: eg = evergreen, sg = summergreen, hc = hemicryptophyte, p = perennial. (DOCX) [file pone.0111023.s003.docx]

Table S1. Species characteristics (Jäger & Werner 2005) and coordinates of the localities where seeds were collected. Growth form: eg=evergreen, sg=summergreen, hc= hemicryptophyte, p= perennial.

| Genus | Species | Range-type | Growth form | Habitat (in Germany) | Seed origin | |
| --- | --- | --- | --- | --- | --- | --- |
|  |  |  |  |  | Latitude | Longitude |
| *Carlina* | *Vulgaris* | Oceanic | eg, semi-rosulate, hc, hapaxanth | silicate and semidry grasslands, montane - subalpine meadows, cleared forests | 51.547828° N | 11.946354° E |
|  | *Biebersteinii* | Continental | eg, semi-rosulate, hc, hapaxanth | silicate and semidry grasslands, dry – moderately dry ruderal area, cleared forests and forest edges | 48.805876° N | 16.646231° E |
| *Centaurea* | *Scabiosa* | Oceanic | sg, semi-rosulate, hc, p | calcareous dry and semidry grasslands, dry meadows and shrubland | 51.528795° N | 11.889641° E |
|  | *Stoebe* | Continental | eg, semi-rosulate, hc, biennal, hapaxanth | dry and sandy, partly ruderal xerothermic grasslands | 51.503299° N | 11.945023° E |
| *Dianthus* | *Deltoides* | Oceanic | semi-eg, semi-rosulate, p | xerothermic grasslands, dry slopes and edges of forests | 52.510644° N | 11.180309° E |
|  | *Carthusianorum* | Continental | eg, no rosulate, chamaeophyt/ hc, p | dry and sandy grasslands, rare in arid environments | 51.533204° N | 11.981404° E |
| *Inula* | *Conyzae* | Oceanic | eg, semi rosulate, biennal, hapaxanth/short-lived | semidry grasslands, dry shrubland, forests and their edges | 50.963212° N | 11.596487° E |
|  | *Hirta* | Continental | sg, no rosulate, hc, p | xerothermic grasslands, dry slopes and borders of forests | 51.534788° N | 11.902825° E |
| *Koeleria* | *Pyramidata* | Oceanic | sg, hc, p | dry and semidry grasslands, dry ruderal areas, cleared pine-forests | 51.592711° N | 9.949324° E |
|  | *Macrantha* | Continental | sg, hc/ geophyt, p | dry and semidry grasslands, dry ruderal areas, dry meadows, cleared pine-forests | 51.532948° N | 11.914366° E |
| *Scabiosa* | *Columbaria* | Oceanic | eg, semi-rosulate, hc, short-lived, p | calcareous dry and semidry grasslands, meadows and shrubland | 51.592711° N | 9.949324° E |
|  | *Ochroleuca* | Continental | eg, semi-rosulate, hc, p | continental, dry and semidry grasslands | 51.533204° N | 11.981404° E |
| *Silene* | *Nutans* | Oceanic | sg, semi-rosulate, hc, p | silicate dry grasslands, dry shrubland, dry forests and their edges | 51.592302° N | 9.948576° E |
|  | *Otites* | Continental | eg, semi-rosulate, hc, short-lived, p | calcareous, silicate and sandy dry grasslands, dry pine-forests | 51.527867° N | 11.890091° E |

Jäger EJ, Werner K, editors (2005) Werner Rothmaler: Exkursionsflora von Deutschland. 10. edition. München: Elsevier (Spektrum)
